# A Stakeholder Analysis of Schistosomiasis Diagnostic Landscape in South-West Nigeria: Insights for Diagnostics Co-creation

**DOI:** 10.3389/fpubh.2020.564381

**Published:** 2020-10-30

**Authors:** Adeola Onasanya, Maryam Keshinro, Oladimeji Oladepo, Jo Van Engelen, Jan Carel Diehl

**Affiliations:** ^1^Department of Sustainable Design Engineering, Faculty of Industrial Design Engineering, Delft University of Technology, Delft, Netherlands; ^2^Department of Parasitology, Leiden University Medical Center, Leiden, Netherlands; ^3^Department of Health Promotion and Education, Faculty of Public Health, College of Medicine, University of Ibadan, Oyo, Nigeria

**Keywords:** schistosomiasis, stakeholders, co-creation, diagnostics, power, interest

## Abstract

**Background:** Schistosomiasis, one of the neglected tropical diseases, is a water-based parasitic disease of public health importance. Currently, tests for *Schistosoma haematobium* infection either demonstrate poor specificity, are expensive or too laborious for use in endemic countries, creating a need for more sensitive, cheaper, and easy to use devices for the diagnosis of schistosomiasis. To ensure engagement during the process of device development; and effective acceptance and use after the introduction of diagnostics devices for *S. haematobium*, there is a need to involve stakeholders with varying power, interest, and stakes in device co-creation, as well as those relevant for later use situation in the diagnostic landscape. The main goal of this study is to identify and analyze relevant stakeholders for co-creation using a power-interest matrix.

**Materials and Methods:** The study was based on an action research methodology using a case study approach. A contextual inquiry approach consisting of 2 stages: stakeholder identification and interview; and stakeholder analysis was used. The field part of the study was carried out in Oyo State, Nigeria using a multistage cluster purposive sampling technique based on the category of stakeholders to be interviewed predicated on the organizational structure within the state and communities. A mix of qualitative research techniques was used. Identified themes related to power and interest were mapped and analyzed.

**Results:** We identified 17 characteristics of stakeholders across 7 categories of stakeholders important for schistosomiasis diagnostics. Most of the stakeholders were important for both the co-creation and adoption phase of the device development for diagnostics. However, not all stakeholders were relevant to co-creation. Key Stakeholders relevant for diagnostics co-creation demonstrated significant social power, organization power, and legitimate power bases. Most of the stakeholders showed significant interest in the device to be created.

**Discussion:** The power and interest of these stakeholders reveal some insight into how each stakeholder may be engaged for both co-creation and device usage. The involvement of relevant actors who will also be important for co-creation and implementation, will simplify the engagement process for the critical stakeholders, increase the ability to manage the process, and increase diagnostic device acceptability.

## Background

Schistosomiasis, one of the 20 Neglected Tropical Diseases (NTDs), is a water-based parasitic disease of public health importance. The disease, which currently affects over 250 million people, is endemic in Sub-Saharan Africa (1, 2). There are five different types of species causing schistosomiasis infection: *Schistosoma haematobium* affecting the urinary tract; *Schistosoma mansoni, Schistosoma japonicum, Schistosoma intercalatum*, and *Schistosoma mekongi* affecting the intestine. *S. haematobium* and *S. mansoni* infections are common in Africa (2, 3). Of these species, *S. haematobium* is the most prevalent parasite in Nigeria affecting an estimated 30 million people yearly (1, 4). *S. haematobium* infection is endemic in many rural and agrarian communities in Nigeria that interact with water through subsistence farming, fishing, washing activities, and water recreational activities (5, 6). The constant contact with water containing *S. haematobium* cercariae released from the Bulinus snail, which occurs regularly, often results in re-infection with the disease, and this impacts on the data on disease prevalence (1, 3–5). Since adult worms have been documented to live in humans for as long as 30 years, most long-term residents of endemic areas become infected or re-infected with schistosomes at some point in their life (7) leading to a vicious cycle within the communities. Besides, depending on the stage of the infection, a wide range of clinical symptoms may occur, many of which are hard to distinguish from several other diseases (5). However, it is a notable cause of morbidity with many infected persons experiencing hematuria, dysuria, bladder-wall pathology, and hydronephrosis (8). Although Nigeria has one of the largest schistosomiasis disease burdens in the world, currently, there is no accurate national data on the prevalence of the disease (1). While the country currently undertakes a large-scale deworming exercise of school-age children in endemic zones with praziquantel (9), addressing diagnosis among adults who are not covered by mass administration of praziquantel is a challenge to the disease control.

Nigeria currently tackles schistosomiasis through a 2-step approach: case management and a control program (1, 10). In the case management approach, cases are diagnosed at the primary care level. For the control program, Nigeria has a schistosomiasis control program wherein school-aged children are given praziquantel for the treatment of schistosomiasis. Schistosomiasis is common among children with the highest intensity of infection found in children between ages 5 and 15 years (11), but it is also known that women and men carry a high risk of urinary schistosomiasis due to social and occupational activities such as farming and washing, especially in areas with poor water, and sanitation services (1). In this respect, there are concerns about missed diagnosis for several reasons. First, several persons do not pass bloody urine which is characteristic of the disease (12). Second, the current control program does not include adults in mass drug administration (1, 9) which means that several adults are likely to have schistosomiasis and are not being treated. Third, *S. haematobium* infection is mainly diagnosed currently using microscopy to detect parasite eggs in urine specimens which is not sensitive in detecting light infections of <50 eggs per 10 mls of urine (13), is labor-intensive, and sensitivity of diagnosis depends on the skill of the laboratory personnel (5, 6, 12). Also, egg excretion in urine varies daily and can be complicated by interaction between the host and the parasite (14). Other tests for detecting *S. haematobium* infection either demonstrate poor specificity, high cost, or painstaking logistics for use in endemic countries (6, 15). Besides, some of these tests are more useful during the elimination phase of the control which has not been reached by a large number of countries (16). As such, there is a need for more sensitive, cheaper, and easy to use devices for the diagnosis and control of schistosomiasis.

To address these issues, the project INSPiRED—INclusive diagnoStics for Poverty RElated parasitic Diseases in Nigeria and Gabon, was initiated to explore ways to create a new device for the diagnostics of *S. haematobium* infection within the context of countries with a high disease burden such as Nigeria using a human-centered approach. The project aims to design easy to use, affordable, and reliable diagnostics devices which may deliver the most effective and efficient step toward schistosomiasis control, aligned with the country's model of care. The device to be co-created is a smart optical device for the diagnosis of schistosomiasis (17) which will be developed from a sustainability point of view and not a profit point of view (18, 19). We regard sustainability in the context of ecological, financial, and social consequences of the device to the society which is most visible through a continuous process of improvement exemplified by the co-creation process (20). The devices will eventually be locally manufactured using locally available materials and components. This will reduce the cost of production, reduce dependence on imports, will enable local and maintenance, and contribute to the economy of countries that are willing to adopt the device.

A crucial first step in the designing of the new device is the proper understanding of the schistosomiasis diagnostics landscape in the context of use for several reasons. First, prompt, accurate, and timely diagnosis is important for schistosomiasis control. Since treatment with praziquantel is cheap and readily available, easy to use diagnostics appears to be critical to schistosomiasis control.

Second, a diagnostic device is only effective if it is designed for its context, and this context is complex and deserves an in-depth study. In this situation, several factors such as the people, process, technology, customer requirements, and innovation need to be addressed (21) through multi-stakeholders input at all stages of development, testing, evaluation, and advocacy for adoption. This implies that stakeholders need to give insight into the process and context-of-use of the technology, including device requirements and the innovation context. The alternative to this co-creation process is a top-down approach focusing on the technology itself which has been reported to have limited successful outcomes due to variation between contexts of use and the design context (22). Besides, the complexity of the context, in this case, the social and healthcare context, cannot be detected in detail from a distance. Since the social context is a critical influencer of the stakeholders' outlook, the stakeholders within this social context can be viewed as both static in terms of the operational arrangement of stakeholders (network structure); and dynamic in terms of stakeholder roles, interactions, flows, and interdependencies (23, 24) which have to be taken into consideration during co-creation. Since stakeholders also vary in background, power, interest, and stakes; the complexity of the stakeholder co-creation process must be effectively managed to achieve the expected outcome. Consequently, there is a need to understand how the actors or stakeholders in this context interact through both stakeholder identification, and understanding of the stakeholder network structure and dynamics. As such, it is important to involve stakeholders in the entire device development process.

Third, there is a multiplicity of stakeholders with this context. Initially influencing and involving them in designing the new diagnostic device seems to be a proper approach to co-creation (23–26). Co-creation has a large role to play in the generation of new knowledge and ideas, development of new insights into existing interventions, and concept development (27, 28). To ensure that diagnostic devices are useful in the context for which they are created, it is critical to involve end-users and other important stakeholders through the entire co-creation process. Such involvement will likely lead to increased uptake of the created product. It has been reported that stakeholders perceive a sense of ownership through active participation in the development of diagnostic devices leading to a more efficient solution to achieving positive societal changes (29). To co-create a robust solution, there is a need to identify the stakeholders who are likely to interact with the product. Identification of stakeholders who are important for this process, and understanding their characteristics can help address the gaps and challenges that can impact on device development. Besides, the fact that stakeholders have different views on the problem of schistosomiasis diagnostics and differing solutions means that stakeholders will have different important insights to contribute (30). Although it appears that the government is the most visible stakeholder, it is important to note that other stakeholders such as health workers and patients can impact on the design and use of a diagnostic device for schistosomiasis.

After the stakeholder identification, it is important to analyze the stakeholders using key characteristics that are useful during the process and life cycle of device development. Stakeholder analysis is a process that defines aspects of a phenomenon affected by a decision or action, identifies individuals, groups, and organizations affected by or that can affect those parts of the phenomenon; and prioritizes these individuals and groups for involvement in the decision-making process (31). Stakeholder analysis is useful for assessing their knowledge about the schistosomiasis diagnosis as well as their interests and power. Consequently, our study aims to describe how to effectively identify, select, and analyze important stakeholders for co-creation, as well as identify potential stakeholders for the adoption and implementation of a schistosomiasis device for large scale use.

Although there exists a need to involve important stakeholders when addressing the schistosomiasis diagnostic landscape, there is little information on the required techniques to do so (30). Moreover, in the field of NTDs, it appears that there are no studies on the involvement of stakeholders in the co-creation of a device or the context for design specifically for *S. haematobium*, to the best of our knowledge. There are, however, several studies on NTDs that involve stakeholder analysis (32–38). For these studies, stakeholders are usually identified through purposive sampling (32–35, 37). Most of the studies involved stakeholders at the macro-level (32, 34, 37, 38) with a few studies involving stakeholders at the community level (33) or both (35). However, using purposive sampling alone for stakeholder identification means that some stakeholders who are not in the same network with the identifying stakeholders might be missed.

We also did not find any framework for stakeholder identification and analysis fully tailored for NTD research. Also, we did not find any guidelines or frameworks for the co-creation of diagnostic devices for schistosomiasis. In this paper, we will present a framework for stakeholder identification based on our understanding of the healthcare system and schistosomiasis context in Nigeria, and a contextual inquiry framework (39) used by Van Woezik et al. (30). We will present the results of applying this framework to a stakeholder identification process during the process of co-creation of services, devices, and policy with stakeholders. We will also present our analysis of relevant stakeholders' power, interest, and stakes for device co-creation using a power-interest matrix. We will close the paper by discussing how such a strategy might help to identify relevant stakeholders within a specific field of study and to develop ways of engaging and co-creating with stakeholders based on the outcome of the analysis.

## Methods

The study used an action research methodology with Oyo State, Nigeria, as a case study. Qualitative data collected include key informant interviews, in-depth interviews, focus group discussions, expert recommendations, and document analysis. The qualitative method of data collection is rich and reveals the complexities and the depths of what can be abstracted for stakeholder analysis.

### Research Approach

We used a contextual inquiry approach, similar to work done by Van Woezik et al. (30). This consists of 2 stages: stakeholder identification and interview; and stakeholder analysis using the qualitative data to create a power-interest matrix.

### Stakeholder Identification

**Figure 1:**
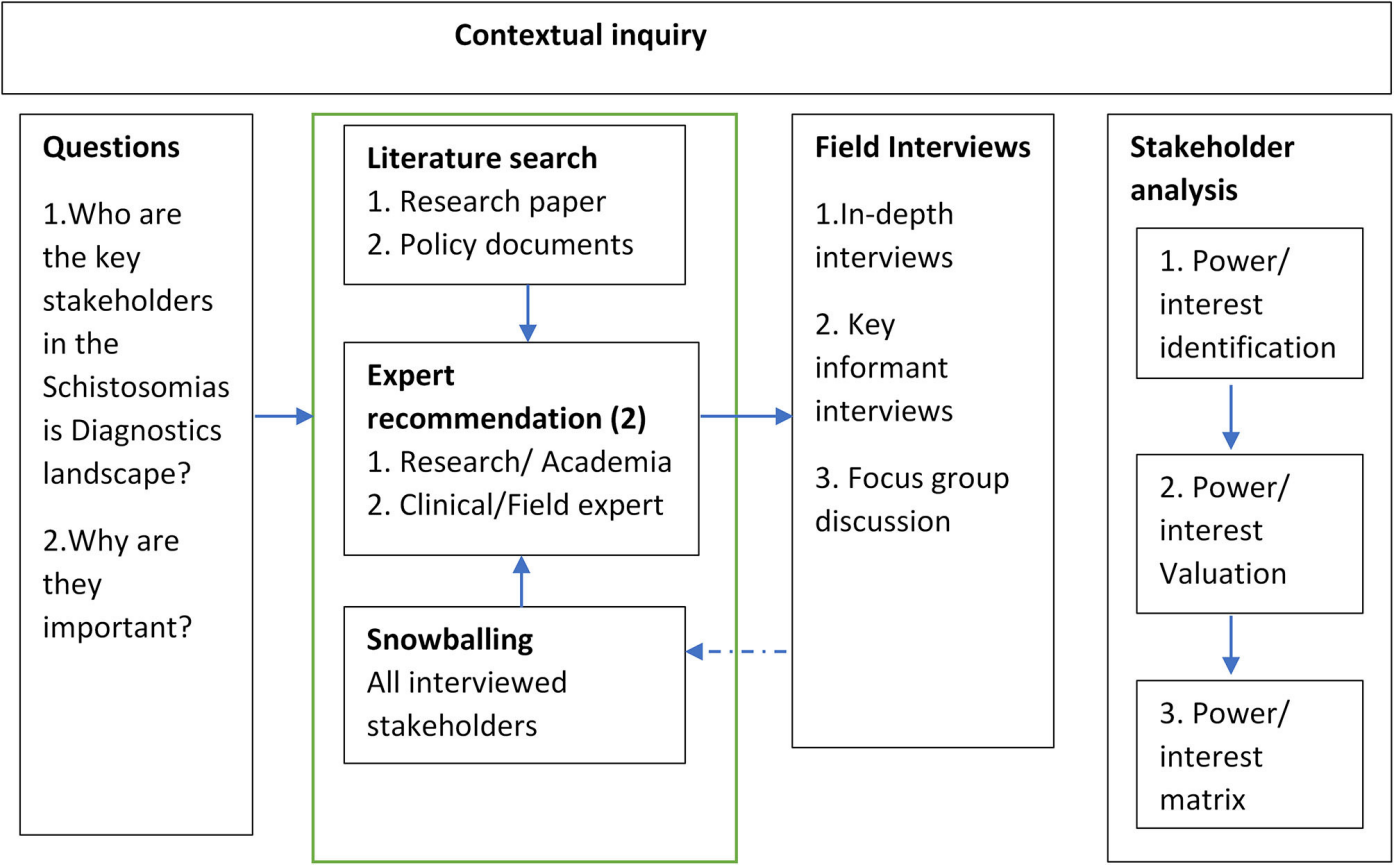
Process map of contextual inquiry into the schistosomiasis diagnostics landscape.

We defined a stakeholder as any person, group, or organization who should be or is involved in schistosomiasis diagnosis based on Freeman's definition of a stakeholder (39). The first stage of the process consists of 3 levels of inquiry using a mixed approach into the context of stakeholders important to the research (Figure 1).

1. Literature scan: First, we identified stakeholders based on the literature on NTD research (4, 40–42) as well as policy documents on schistosomiasis in Nigeria (9, 11).
2. Expert panel recommendation: After identifying stakeholders from literature, we involved 2 experienced experts from public health research and clinical medicine, respectively to validate and make suggestions on other stakeholders who were important to the diagnostic landscape in Nigeria.
3. Snowballing: We used a snowballing technique in which we asked all interviewed stakeholders to identify other stakeholders that might be important to schistosomiasis diagnosis in Nigeria.

The outcome of the first two steps of the contextual inquiry process led to the creation of stakeholder categories based on the conceptualization of the demand and supply aspect of healthcare diagnostics for schistosomiasis using stakeholder characteristics (Table 1). The final list of interviewed stakeholders was validated through a 2-step process. First, all identified stakeholders were selected based on meeting at least 3 of the following criteria which were developed from the research question in Figure 1: (1) suggestion by experts and/or stakeholders, and/or literature (2) having a clear stake in schistosomiasis diagnostic landscape, and/or (3) being a potential end-user of the to-be-developed diagnostic device, and or (4) having a strong influence on the demand of the to-be-developed diagnostic device. Second, the generated list was finally reviewed by 2 experts from public health research and clinical medicine, respectively using a binary approach of Yes/No. The final stakeholder categories of stakeholders and a list of important stakeholders were agreed upon by all members of the team.

**Table 1:** Stakeholder categorization for diagnostics co-creation.

**Category**	**Characteristics**
1	Persons/parents of children who have been previously diagnosed and or treated for *S. haematobium* infection within the last 3 years.
2	Stakeholders within the community that can impact the patient's decision to access care (diagnostics, and or treatment).
3	Stakeholders within the formal health system (both public and private) who can diagnose and or treat patients with schistosomiasis.
4	Stakeholders within the government who are in charge of programs/processes to identify, and or treat schistosomiasis.
5	Stakeholders in Non-Governmental Organizations (NGOs) that contribute to schistosomiasis diagnosis, and or treatment within the state.
6	Stakeholders in academia who are working in the Neglected Tropical Disease field.
7	Stakeholders that finance diagnosis and or treatment of Neglected Tropical Diseases.

### Study Setting and Sampling Approach

Based on stakeholder categories in Table 1, the field part of the study was carried out in Oyo State, South-West Nigeria. The state has a moderate prevalence of schistosomiasis infection (1, 4). For category 1–3 stakeholders, we used a multistage cluster purposive sampling technique. Two local government areas (LGA); urban and rural, respectively were selected based on ecological factors such as closeness to rivers which were known reservoirs of *S. haematobium* infection. One ward from each local government structure was also selected based on ecological factors. Based on information available from the local governments on recently treated schistosomiasis cases (category 1 stakeholder), we selected and interviewed category 2 and 3 stakeholders based on geographical proximity to the area of residence of category 1 stakeholders. Category 4–6 stakeholders were sampled using purposive sampling. The sample size is difficult to determine a priori because of the explorative nature of this research. However, our final sample size was considered sufficient when it met the following criteria: (1) a minimum of 30 interviewed stakeholders based on recommendations by Marshall et al. (43); (2) when theoretical saturation is reached by no new mention of stakeholders from the snowballing technique. A respondent was considered a good fit when he/she met the criteria in Table 1 and was validated by the 2-step process described earlier.

### Stakeholder Interview and Analysis

We carried out qualitative (In-depth and Key informant) interviews and Focus Group Discussions (FGD) with stakeholders. The questions asked depends on the stakeholder background and experience. However, questions asked include normative ideas on *S. haematobium* infection, interaction with formal and informal health care, current diagnostic landscape, and diagnostic challenges and limitations. Consent was given before the interviews. Ethical approval for the study was obtained from the Ethical Review Committee of the College of Medicine, University of Ibadan, Nigeria (NHREC/05/01/2008a).

Interviews were transcribed and translated where applicable. Transcripts were reviewed by two researchers, entered into atlas.ti version 8.4 and coded using the deductive thematic analysis method. A researcher coded the interviews and created a coding manual. Two other researchers validated this.

All researchers within the team independently validated the identified themes related to power and interest. Power was defined as “the level of influence a stakeholder has in the diagnosis of *S. haematobium* infection” (30). The sources of power could include: political, economic, social, cultural, historical, and/or organizational factors (26, 44). The expression of these sources of power (power bases) includes reward, coercion, information, legitimate, expert, and referent which can be derived from political, economic, social, cultural, historical, and/or organizational factors (26, 44). Interest was defined as “value abstraction to the new diagnostic device for the diagnosis of *S. haematobium* infection” (45). Interests could either be “expressed” or “implied/ manifested” in direction and willingness-to-use magnitude (46).

Based on the results of the analysis, stakeholders were further categorized into four levels of analysis of stakeholders based on the four-level model of the healthcare system, which was adapted from Reid et al. (47). The themes were analyzed based on the level in which stakeholders fall into. Thereafter, stakeholders were ranked based on their power and interest, which were valued on a scale of 1–5, with 1 meaning low level and 5 meaning the highest level of power and interests, respectively similar to the ranking by Hyder et al. (48). The results of these analyzed stakeholders were mapped to identify stakeholders who were important to co-creation into players, context setters, crowds, and subjects (49).

## Results

### Stakeholder Characteristics

A total of 17 stakeholder characteristics were identified across the 7 categories (Table 2). This yielded a total of 36 stakeholders to be interviewed. Thirty three stakeholders were interviewed. One stakeholder (religious body) was not interviewed based on the large variance in types and modes of operation of religious bodies, 2 other stakeholders (State Disease Surveillance and Notification Officer (DSNO) and Federal NTD officer) were not available for interviews.

**Table 2:** Stakeholder characteristic and identification for co-creation.

**Stakeholder category**	**Role**	**Method of identification**	**Number interviewed**
1	Parent/guardian of children with schistosomiasis	Literature review	5
2	Community leader	Expert	1
	Patent Medicine Vendor (PMV)	Expert	1
	Traditional healer	Expert	1
	Community mobilizer	Snowballing	1
3	Doctors	Literature review	1
	Community Health officers	Snowballing	1
	Laboratory scientist/Technician	Literature review	5
	Community Health Extension Workers (CHEW)	Literature review	2
4	Primary health care (PHC) coordinator	Literature review	2
	NTD officer	Literature review	3
	Disease surveillance and notification Officer (DSNO)	Snowballing	2
	Teachers	Snowballing	2
5	NGO	Literature review	1
	Community-based organization (CBO)	Expert	0
6	Academia	Literature review	3
7	Financing	Expert	1

^*^One interview was an FGD.

As can be seen from Table 2, the literature scan identified 6 stakeholder characteristics, 5 stakeholder characteristics were identified by experts and by snowballing, respectively.

**Figure 2:**
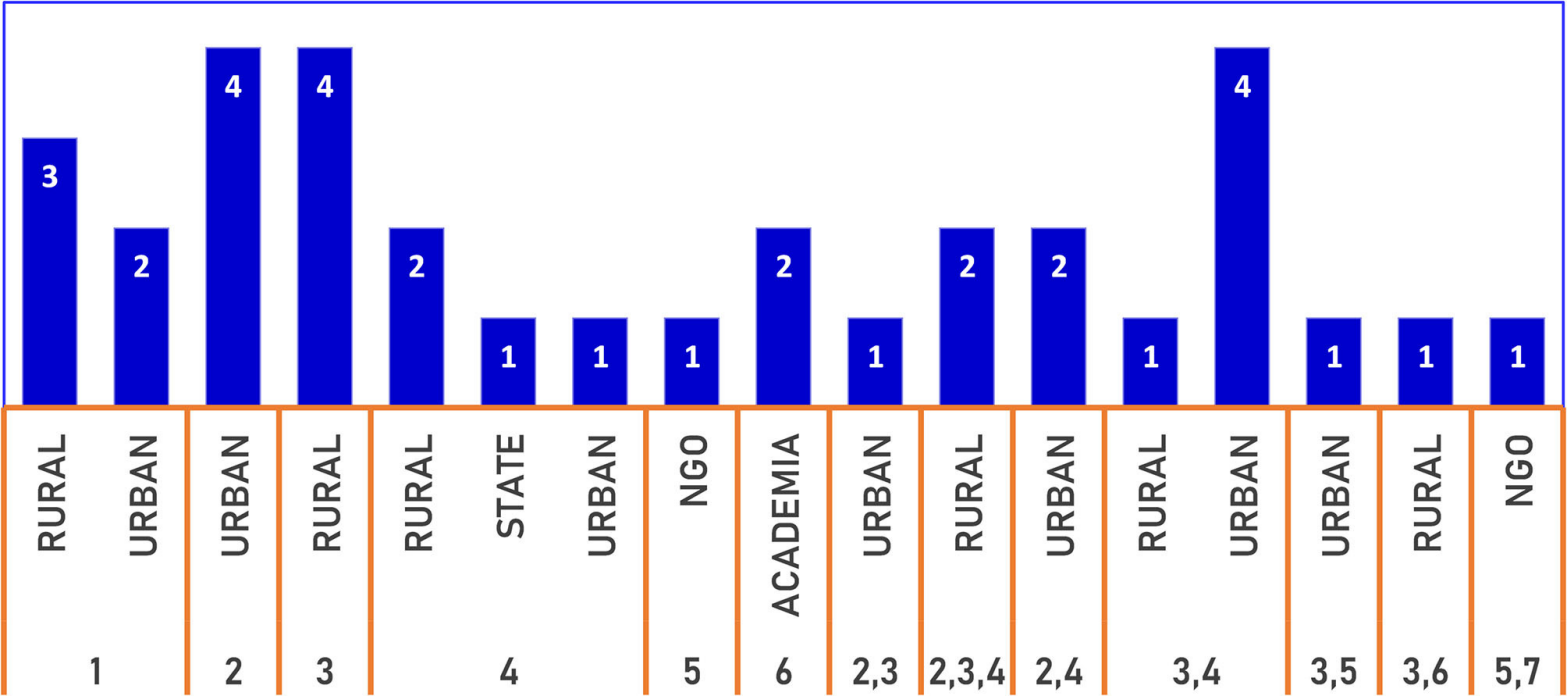
Stakeholder characteristics.

Figure 2 has a breakdown of the number of stakeholders according to the location. Twenty stakeholders performed a singular role, 13 stakeholders performed 2 roles, while another 2 stakeholders performed 3 roles concurrently. At the local government level, the location of the community (rural or urban) did not significantly determine the multiplicity of roles.

**Figure 3:**
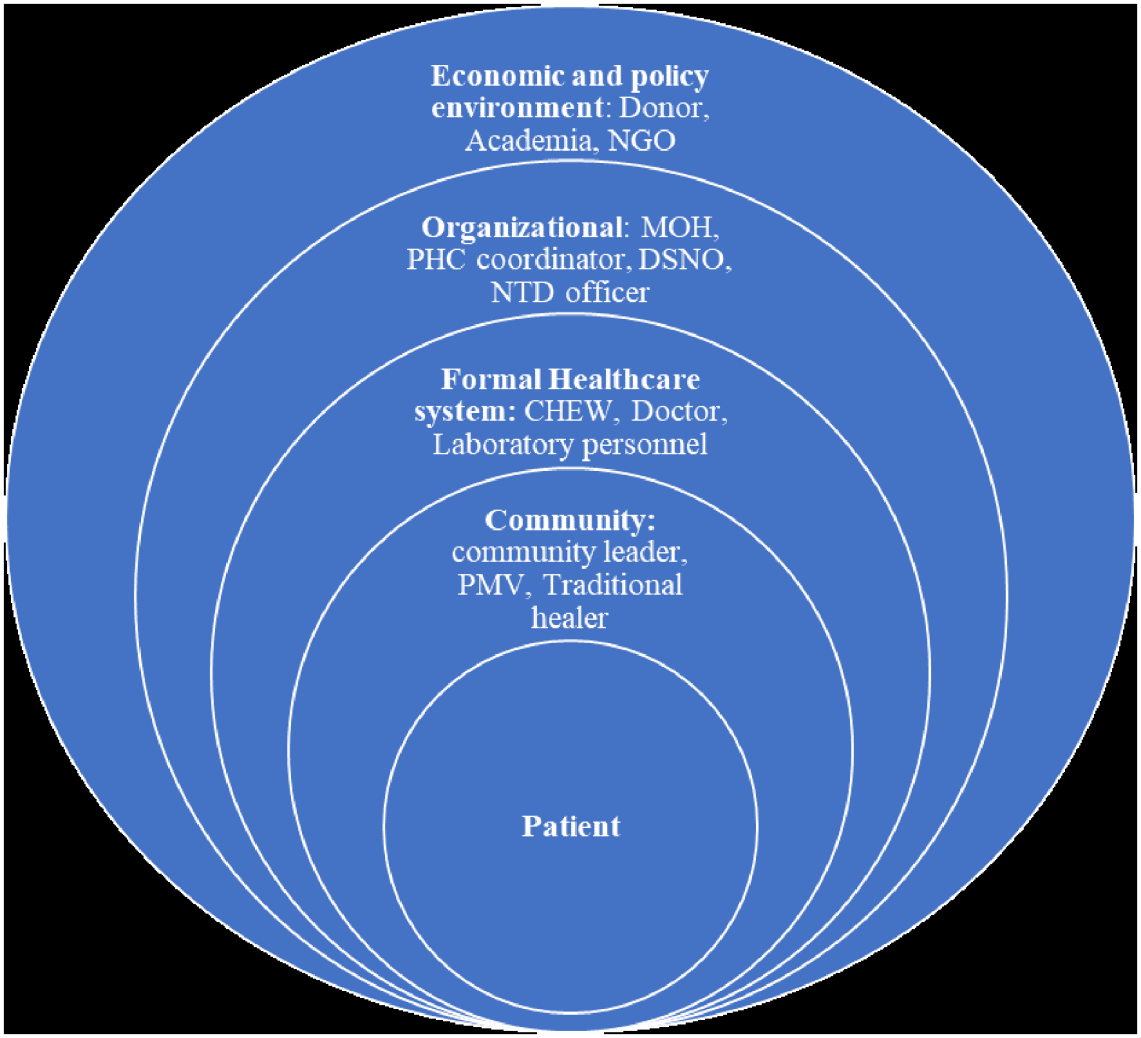
Stakeholder categorization within the health system.

Stakeholders' power and interest in schistosomiasis diagnostics were further analyzed by categorizing stakeholders into four levels which were adapted from Reid et al. (47). Based on this level of analysis (Figure 3), Stakeholder categories 1–2 falls within the micro-level or community level, stakeholders within category 3 fall into the health care level; stakeholders in category 4 fall within the organizational level and category 5–7 stakeholders fall into policy/economic environment. Some stakeholders fall within 2 or more categories based on their multiple roles. Stakeholders relevant for diagnostics co-creation had significant social power, organization power, and legitimate power bases at each level of analysis. All stakeholders were influenced both by other stakeholders within their level and by the next level of stakeholder within the lower and higher concentric circle (Figure 3).

We also found stakeholders that were important for both co-creation and adoption of technology. Although the initial focus was on diagnostics co-creation, we were also able to identify some stakeholders from the interview transcripts who did not fall into the diagnostic co-creation categories but may be important for the adoption of the device based on the 2-step validation process for all stakeholders. However, these do not show the complete extent of stakeholders for adoption and implementation (Table 3).

**Table 3:** Stakeholder characteristics and stage of device lifecycle.

**Characteristics**	**Stage of the device development lifecycle**
Parent/guardian of children with schistosomiasis	Implementation/adoption
Community members	Co-creation and Implementation/adoption
Community leader	Co-creation and Implementation/adoption
Patent Medicine Vendor (PMV)	–
Traditional healer	–
Market associations	Implementation/adoption
Community health committee	Implementation/adoption
Community mobilizer	Co-creation and Implementation/adoption
Doctors	Co-creation and Implementation/adoption
Community Health officers	Co-creation and Implementation/adoption
Laboratory scientist/Technician	Co-creation and Implementation/adoption
Community Health Extension Workers (CHEW)	Co-creation and Implementation/adoption
Primary health care (PHC) coordinator	Co-creation and Implementation/adoption
Primary health care board director	Implementation/adoption
NTD officer (State and LGA)	Co-creation and Implementation/adoption
Disease surveillance and notification Officer (DSNO) (State and LGA)	Co-creation and Implementation/adoption
Teachers	–
NGO	Co-creation and Implementation/adoption
Community-based organization (CBO)	Co-creation and Implementation/adoption
Academia	Co-creation and Implementation/adoption
Financing	Co-creation and Implementation/adoption
Biomedical Engineer	Co-creation and Implementation/adoption
National Center for Disease control	Implementation/adoption
Media	Implementation/adoption
Politicians	Implementation/adoption
Equipment suppliers	Implementation/adoption
Federal Ministry of Health	Co-creation and Implementation/adoption
World Health Organization	Co-creation and Implementation/adoption

### Stakeholder Power/Influence Thematic Analysis

All the important stakeholders that were interviewed, demonstrated varying types and levels of power. A summary of this can be found in Table 4.

**Table 4:** Stakeholder power and interest ranking.

**Stakeholder category**	**Role**	**Power type**	**Power rank**	**Interest rank**
1	Parent/guardian of children with schistosomiasis	Social, coercion	2	2
2	Community leader	Social, legitimate	2	1
	Patent Medicine Vendor (PMV)	Social, referent	2	1
	Traditional healer	Social, cultural	2	1
	Community mobilizer	Social, informational, referent	3	3
3	Doctors	Expert, referent	3	3
	Community Health officers	Expert, referent	3	3
	Laboratory scientist/Technician	Expert, referent	3	4
	Community Health Extension Workers (CHEW)	Social, Expert, referent	4	5
4	Primary health care (PHC) coordinator	Organizational, Expert, legitimate	3	3
	NTD officer	Organizational, informational, social	3	3
	Disease surveillance and notification Officer (DSNO)	Organizational, legitimate, Expert, social, informational	4	4
	Teachers	Informational	1	1
5	NGO	Organizational, legitimate, informational	3	3
	Community-based organization (CBO)	–	–	–
6	Academia	Expert, informational	3	5
7	Financing	Organizational, informational	5	4

### Community-Level Stakeholders

Community-level stakeholders demonstrated varying levels of power. These stakeholders consist of individuals: patient's parents/guardians, traditional healer, community leader, community mobilizer, and Patent Medicine Vendor (PMV), all embedded within the same community network.

### Patient

All the patients or their guardians individually did not demonstrate any significant power. However, collectively, they have a great source of social power which determines the demand for healthcare. The decision to access healthcare was made based on either financial situation, social relationships, trust and or ease of access to the formal health system (CHEW or Doctor) or other sources of healthcare (PMV, traditional healer). This social power is important to drive the use and demand for diagnostics. This power did not significantly differ between rural and urban areas. However, guardians in the urban areas were more likely to use a hospital as a first step than using other sources of treatment.

“*Mummy* (referring to community mobilizer) *asked him to go to the hospital and she also informs his dad because she is closer to him, so they take him to the hospital and he was treated and they ensure that he is okay before he traveled*.” Patient's guardian, male, urban area.

For rural areas, the patient was more likely to take some other steps, before accessing healthcare

“*When that illness started with the child, he was running temperature and we gave him herbs but it was not effective… We gave him paracetamol and yet there was no difference, … later saw him urinate and sighted blood in his urine… We waited for our husband to come back. When he comes back he took him to daddy (referring to CHEW) at… we did not know about the disease, and he took care of it*.” Patients' parent, female, rural area.

### Traditional Healer

The traditional healer demonstrated some degree of power over patients seeking care. Power was based on cultural and social factors. A traditional healer could also demonstrate referent power by referring non-improving cases to the hospital.

“…*so I gave him traditional herbs, they are what we had previous knowledge of and when he drank it, he was cured*.” *Apart from that one, he also brought his boss to me, … we treated his boss with the same herbs we used to treat him. So when he was okay I told him to go for further treatment at the hospital and to check if you are totally cured'* Traditional healer, male, urban area.

The traditional healer also mentioned the patient's autonomy in seeking diagnosis and treatment

“*People in those days (in the past) listen to advice but nowadays people do not take advice anymore*” Traditional healer, male, urban area.

### Community Leader

The community leader demonstrates some form of legitimate power over the community but this power is limited to giving advice. The inherent power source of the community leader may likely impact on power demonstrated as those with cultural/ historical power source may demonstrate more power.

“*so when such a thing occur we normally advise the parents to carry such children to the hospital*” Community leader, male, urban area.

### Community Mobilizer

The community mobilizer demonstrates some form of social power based on relationships and could also demonstrate expert power depending on training.

“*the way we interact, you can see that as I got here now, they started greeting me… because of the relationship I have with them… and clinically we diagnose them I mean we treated them clinically because there is no laboratory to confirm it*” Community Health Officer and Community Mobilizer, male, rural area.

“*yes we serve as the interface between the government and the people of this community, so we usually sensitize them about their health, their environment… those (patients) that can manage to go (to the hospital) without any problem and has an assistant, I ask them to go, and those that are too weak to go by themselves like (an) emergency, I followed them*.” Community mobilizer, female, urban area.

### PMV

The patent medicine vendor's (PMVs) source of power came from social relationships, expertise, and had the power to refer patients to seek care. There are two types of PMVs: mobile and resident. Resident PMVs have more power over the patient's care and access to diagnosis

“*If they are ill and it is body temperature that just started, so I will give hem drugs for two days, sometimes if there is no changes we refer them to the health center*” PMV, female, urban area.

“*I usually greet and ask them about their health when they pass by my shop, sometime some will thank me for the drugs I gave them the day before and that it's effective while another may come to report that the medicine was not effective and request for another kind*.” PMV, female, urban area.

Resident PMVs viewed their power over disease diagnosis to be limited to what was acceptable by law. Due to their presence within the locality, they could easily be identified and liable to the law.

“…*so far it won't affect us, you know there is nothing that the police don't investigate, so if it won't affect us and the police won't disturb us, no problem*” PMV, female, urban area.

### Health Care Level Stakeholders

This level of Stakeholders includes Doctors, Nurses, Community Health Officers (CHO), Community Health Extension Workers (CHEWS), Laboratory Scientists/Technicians. They work within the clinical aspect of the health care system.

### CHEWS/CHO/Doctors

The CHEWS/Doctors are the first level of entry into the healthcare system. Due to the limited number of personnel within the healthcare system, CHEWS/CHOs are in charge of smaller primary health care centers and health posts that are close to the communities, while Doctors were in Charge of larger health centers. The CHEW, CHO, and Doctor demonstrated power as experts. However, the CHEWs also demonstrated social power based on their continuous residence within the community leading to the formation of relationships with members of the community.

“… *I have more information, so they really do not have a lot of options than to follow my instructions; this is not in all cases but it happens most the time…If I tell them that I want to admit them, then they do not have a choice. If they refuse to stay, this is not a prison and they can leave. It depends on how you talk to them anyway*” Doctor, male, urban area.

“.. *(Patient)came into the clinic with complaints and then he followed my boss (Senior CHEW) into town for proper diagnosis where tests were carried out on him. After everything, my boss told me they got drugs and that it was schistosomiasis*” Junior CHEW, Health Center, Rural area

“…*At times if I want to go and I run into people passing by going toward the area with their bikes, they often assist me*.” Junior CHEW, Female, rural area.

“*I think I might have seen about two to three cases (of schistosomiasis). When this happens, the first thing we do after noting their complaints is to refer them to the MOH(medical officer of Health)*.” CHO, female, urban area.

### Laboratory Personnel

Laboratory personnel demonstrated power as experts with technical knowledge. Their power over patients was limited and they only had contacts with patients through a referral from doctors. That did not have power over treatment or what diagnostic test to carry out.

“*Yes, at that point you, whatsoever analysis is requested from the physician, at the end of my own analysis once I see a result, I have that privilege to also recommend…suggestive. So, it now depends on the physician by the time the patient reports back to the physician*” Laboratory scientist, male, NGO, urban area.

“*our job is to analyze the specimen and report. Then the doctor decides on how to act on our result…they get referred by doctors to here from various hospitals. and people come here on their own… But mostly they are referred here by doctors*” Laboratory scientist, male, private lab, urban area.

“*we first go for microscopy and if there is schistosomiasis, we refer them to the doctors for treatment*” Laboratory technician, female, Health Center, urban area.

### Organizational Level Stakeholders

Organizational level stakeholders were those in charge of programmatic parts of schistosomiasis control as well as gathering and using information about schistosomiasis for program planning. These include the Medical Officer of Health (MOH), Primary Health Care (PHC) coordinator, Disease Surveillance and Notification Officer (DSNO), and the Neglected Tropical Disease Officer (NTD) and teachers.

### Primary Health Care (PHC) Coordinator/MOH

The PHC coordinator /MOH is in charge of primary care at the local government levels demonstrated legitimate power because of their position within the organization part of the healthcare system, as well as expertise based on training.

“*by virtue of my position can relate with other line ministries, department, and agencies, international organizations…that want to partner with the local government on health matters to implement any program as far as the health system of the local government is concerned*.” *I get referrals and at the same time, I do refer people, depending on the case that presents itself. My staff can refer patients to me or invite me to manage a case at the facility level'* MOH and PHC coordinator, male, urban LGA.

“*I see to the affairs of the PHC department in general. I also coordinate the staff in terms of their duties, supervise them, and then if there is any need for recommendation for any of them from the state government, I will make those recommendations*” PHC coordinator, female, rural LGA.

However, the level of power of these officers to address schistosomiasis and recommend a line of action is limited by other stakeholders that do not have a direct relationship with schistosomiasis diagnostics.

“*There are enough skilled people outside but the government did not recruit them. I cannot recruit them by myself, they are usually recruited by the State Primary Healthcare Board*.” PHC coordinator, female, rural LGA.

“*It takes a collaborative effort of my office, the office of the political officeholders. The politicians are the ones who initiate policies and they decide if they want to expand and add more to the existing facilities. They determine the felt need of the people in the community that they serve. When they go to the people and the people tell them that they need a healthcare facility, they work on it. Then, they will refer to me. The process goes from top to bottom, it rarely goes bottom-up*.” MOH and PHC coordinator, male, urban LGA.

### NTD Officer

The NTD officer is primarily in charge of the programmatic aspect of the schistosomiasis control. They demonstrate technical power because of their position. They were also in charge of the School-based deworming day targeting school-age children for treatment for schistosomiasis. They also have ties with the community and could leverage social connections within the community.

“*We only currently handle kids from ages 5 to 14, adults are also prone to the risk and we have seen cases of adults passing blood in urine. This is why several adults have been asking when we will carry out a program like this for them. So, it is necessary for both adults and not the children alone… Maybe the next time we have a meeting with the state, we would bring up that they should extend the range of reach to cater for people 15 years and above because they also swim in the rivers and they can end up infecting the ones we've treated if they are not included*” NTD officer, female, urban LGA.

“*We do surveillance. We try as much as possible to pass messages to the community leaders so that they will be aware of it, so whenever they see signs, they will be able to call on me to inform me about the cases, and then, there will be a linkup between myself and the community*.” NTD officer, male, rural LGA.

The State had legitimate organizational power over the schistosomiasis control program. However, the Federal government determined the overall strategies for schistosomiasis control based on policy.

“*because the state does not have the authority to that (address schistosomiasis through policy). It always comes from the federal level. The guidelines we use are from the federal level and our hands are tied without the federal ministry of health*.” *State NTD Officer*.

### Disease Surveillance and Notification Officer (DSNO)

These officers are in charge of monitoring and reporting notifiable diseases including schistosomiasis. They directly work with health facilities and demonstrate strong legitimate power over health facilities, both private and public, and at all levels of care (primary, secondary and tertiary healthcare) within their jurisdiction.

“*We have weekly and monthly reporting. Whenever they see something of such nature such as blood in the urine, they will send a text message notifying me that there is a case of this nature and on monthly basis, they will sum all the activities for the weeks and send it to me. I have a column that indicates schistosomiasis. Whenever such a case has been reported to me, I must go and investigate in all health facilities… I have to contact the higher authority which is the state disease, surveillance officer. Then we go together and make verification*.” DSNO, male, urban LGA.

“*The health workers there will treat the patient and document it. We will then send the record to the state*.” DSNO, female, rural LGA.

### Teachers

Teachers only featured strongly within the treatment aspect of the schistosomiasis control program. They, however, have limited powers overtreatment and no power over the diagnosis of schistosomiasis.

“*we announce it to them that there is deworming, some of them came some did not come to school and some who came like one he was always tapping me that her mother said she should not take any medicine*” *Primary School Teacher*, Urban area.

“*then it depends on the condition if it one that requires an immediate attention. For example, a kid that has a cut and was injured and he is bleeding several of them have been taken to private clinics around here, the principal pay for their treatment, teachers raising money taken to him, to attend to them at that first day…there were children that have been rushed to hospitals by the school, the parents will come, meet them in the hospital where they were taken to so it depends on what happens. that will determine*…” Participant 3, FGD, Secondary school, urban area.

### Policy/Economic Environment Stakeholders

These stakeholders have a wider level of impact and they interphase with more than one level of the health system simultaneously. These include interaction with both the community level, local Government, State Government, and or at the Federal government level. They include academia/researchers, Non-Governmental Organizations (NGOs), financing/donor organizations.

### Non-Governmental Organizations (NGO)

We identified three main NGOs. One of the NGOs [Association of Reproductive and Family Health (ARFH)] worked within disease diagnostics and the second, the World Health Organization (WHO) performed a technical function. While the third (Evidence action) provided technical function as well as funding support. The WHO function appeared to have stronger legitimate powers by performing supervisory roles. The WHO did not have a state-based NTD officer. This was only present at the national level. However, other officers within the state office filled the gap when needed.

“*This may be due to the fact that I do not really look into it but in my supervisions, I have barely seen cases of schistosomiasis…I think the surveillance.is poor for schistosomiasis. With good surveillance system, I think we will easily pick up quite a number of cases. Many of the factors that might predispose to schistosomiasis is present*” WHO state technical officer.

### Financing

Financing appeared to be one of the greatest sources of power. Financiers had legitimate power as well as the power to coerce the state and the federal government to address schistosomiasis diagnostics. One NGO primarily performed some financing activity targeting schistosomiasis control through the school-based deworming exercise. The NGO also has informational power to bring about change.

“*I do not think that schistosomiasis is really prioritized and there is probably no funding line for it. Funding is also a big issue. No matter the charges, the funders have their interest. If they insist that they want to fund a certain disease, other diseases will suffer*.” WHO state technical Officer.

“*we basically provide technical assistance for the government to be able to carry our deworming…It involves anything from policy, advocacy, planning and collection and distribution of the drug, monitoring the program and…so we supply, we provide funding for them, we also provide the technical know-how, working with the state…well, we went to the government to say we would have to work with them to carry out a state-wide deworming program so in a way should I say we initiated it but it's the government program…and we do not, we are not the one that provide the drugs, the drugs are provided by the federal ministry of health, it's a free donation…through WHO and WHO is the source of supply*” Country Director, Evidence Action.

### Academia/Research

We found three persons in Research and who all performed dual roles. Two were both doctors and researchers, while one was as both a researcher and a laboratory scientist. Researchers exhibited powers as experts based on technical knowledge and could identify other stakeholders as well as reach out to these stakeholders. As such, they had some form of informational power.

“…*based on the report we had, what we did was to get the NGOs working in those areas to get to their local health authorities to let them know of the problem of schistosomiasis because the cases found here were actually from the local health authorities who gave us the medications for free*.” Researcher and Doctor, male.

“*I think the program covers all local government what I now do not know is if they've been able to identify some high-risk regions in the state and have intensified program in those regions as compared to the places with low risk,…but I know the program, the NTD program is state-wide thing*” Researcher and Doctor, female.

“*I want to talk about one, political will, because there are a lot of politics that go around which—you have planned something and because of one thing you don't they just stop it all of a sudden*.” Researcher and Laboratory scientist, male.

### Stakeholder Interest Thematic Analysis

Most of the interviewed stakeholders were interested in the device and its use for the diagnosis of schistosomiasis. Table 4 shows the grading of their level of interest.

### Community-Level Stakeholders

Members of the community did not show a strong interest in the device due to a lack of understanding of how the device works, low level of awareness of the disease, and also because they looked up to the health workers to make certain decisions about diagnosis and treatment. However, other stakeholders were able to give insight into the patients' perspectives on this device.

“*yes, you need sensitization because if you don't sensitize them, they will not know the value of this*” CHO and Community Mobilizer, female, rural area.

“*If the government provides equipment that can bring out result instantly*” Guardian, female, urban area.

### Healthcare Level

Medical personnel appeared to be interested in the device improving the diagnostic process and increasing efficiency, especially in hard to reach areas.

“*I think that's a good idea, and it will be a good development like in the case of malaria…so, it's just a welcome idea*” CHO and Community mobilizer, rural LGA.

“*I know that you people are always moving forward, so I look forward to whatever advances you can make you know to make life easy for us here*” Laboratory scientist, Private lab.

“*If such a device is brought to this healthcare facility, I think it will be easier for us to diagnose patients if such a case is brought to us*.” CHO, Health center, urban LGA.

### Organizational Level

At the organizational level, the PHC coordinator and NTD officers were interested in the device easing workflow and improving diagnosis, thereby helping their output.

“*If you can innovate one that can be appropriated for the ease of local use without microscopy, it will be good since it will be something easy to work with*” PHC coordinator and MOH, male, urban LGA.

*Yes. This is because some will not give you the consent to take their children's urine. We need to convince them totally before samples can be taken…Connecting with the DSNO and going to the UCH (tertiary hospital) takes a very long time. The result also takes time to arrive. It will be better if the diagnosis is done at the PHC level*. PHC coordinator, female, rural LGA.

For the NTD officers, the introduction of the device would increase the effectiveness of their work and reduce waiting times for the conformation of cases from secondary and tertiary hospitals.

“*There is no machine. We do have labs but we are limited to some tests to be carried out at the LGA level. We have to take the samples to UCH (tertiary hospital) to test if it is schistosomiasis…We have lab scientists at the LGA now but the materials they need are not available. If there are materials and equipments to use, they should be able to work*” NTD officer, male, rural LGA.

“*It should let us know people that are coming down with schistosomiasis…*” NTD officer, male, urban LGA.

### Policy/Economic Environment Level

At this level, all stakeholders were interested in the device improving schistosomiasis diagnosis and reducing the impact of disease within the state.

“*Diagnosis is key. For example, tuberculosis control starts with diagnosis before anything can be done. To do this properly, we have to strengthen the labs as the diagnosis and the confirmation of the cure end in the lab. We are advocating point of care devices that could make a diagnosis of some of the public health diseases without a lot of sophistication*” WHO state technical officer.

“*but I think it's.it's potentially a game-changer as to how we do field surveys for Schisto and STH so it's something personally I would really like to get involved in*” Country Director, Evidence Action.

Researchers mentioned the importance of the device's input in quick diagnosis and its importance as a quick screening tool for those with infection or highly endemic regions.

“*They will get the buy-in. If it is for schistosomiasis, the private facilities in places where they have a high burden of that will be interested*” Researcher and Doctor, male.

“*People will embrace it. I'm so sure of that… In fact, already I'm falling in love (with the device)*” Researcher and Laboratory scientist.

“*So if there are better diagnostic test or methods or stuff, that might be able to help so that there are no missing cases, there are obviously missing cases, and I feel that even the few, the ones that we see, they can be picked earlier before it gets to the stage of frank haematuria. They can be picked earlier if we have easy-to-use diagnostic or screening test kit*.” Researcher and Doctor, female.

### Stakeholder Classification and Ranking

Based on the stakeholder power base, and interest evidence available from the interviews, 2 interviewers/researchers read through the transcripts and ranked stakeholders for co-creation according to their power and interest independently (Table 4). Any differences in the ranking were resolved by a more senior researcher.

### Stakeholder Power/Interest Matrix

**Figure 4:**
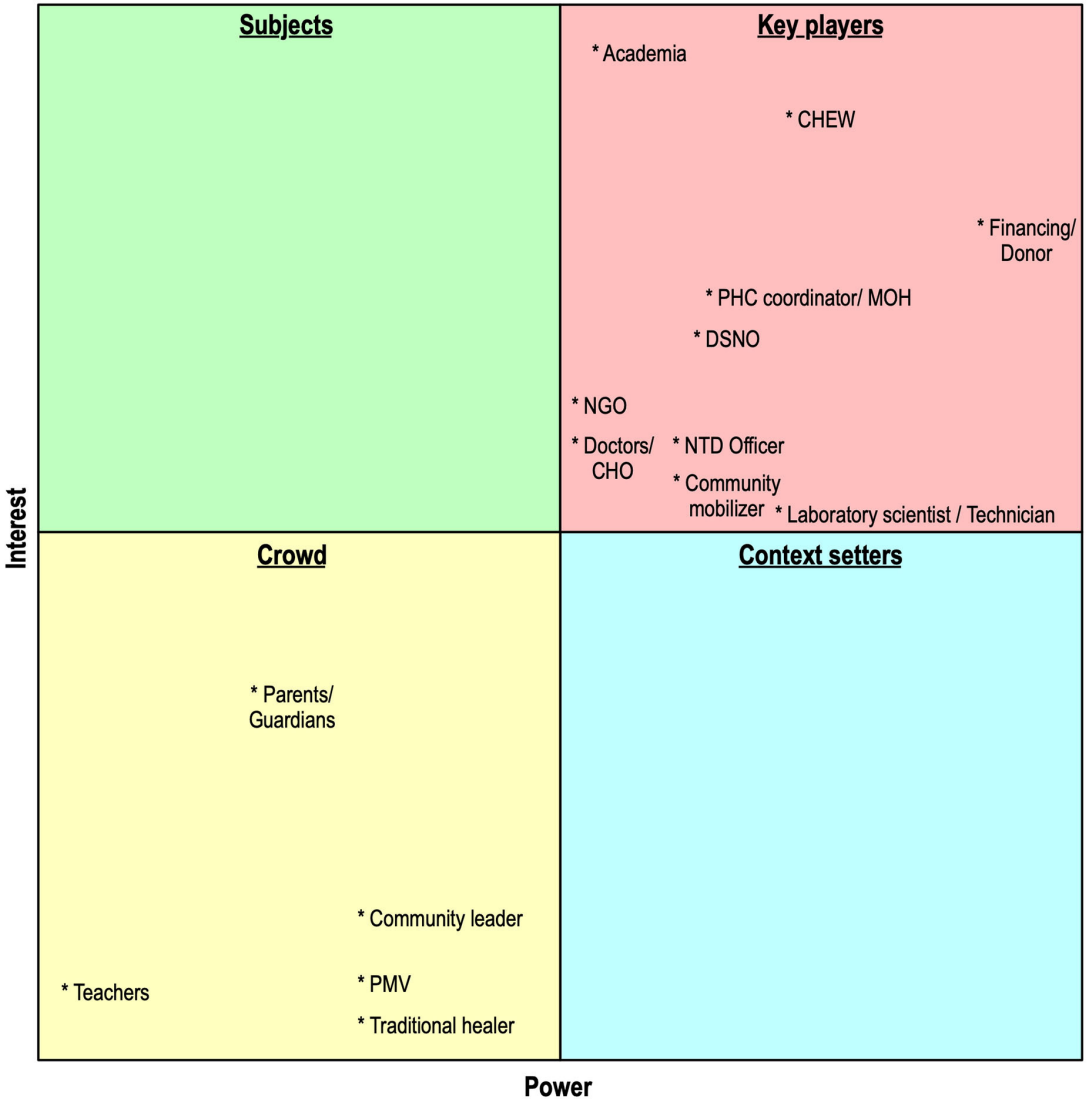
Stakeholder mapping using a power/interest matrix.

Based on the ranking of the stakeholder power and interest, stakeholders for co-creation were mapped into a power interest matrix to identify stakeholders who were important to co-creation. Stakeholders could fall into the following categories (Figure 4): players, context setters, crowds, and subjects (49).

From our analysis, the stakeholders important for co-creation clustered into two categories: “crowd” and “key players.” The “crowd” stakeholders are characterized by low power and low interest. This category is predominantly made up of community-level stakeholders within stakeholder categories 1 and 2. They may have a high impact if they act together toward a goal.

The “key players” stakeholder group consists of category 3–7 stakeholders except for the community mobilizer who falls under category 2 stakeholder. These stakeholders demonstrate high power and high interest. These stakeholders also fall within the organizational, healthcare, and policy/financial environment levels of the healthcare system. Although these players appear to have a high influence/power, these do not necessarily mean high impact since they cannot enforce acceptance by the patients and the community. No stakeholder fell within the category of stakeholders with high interest and low power (subjects) or those with high power and low interest (context setters).

## Discussion

This study assessed and mapped stakeholders' interest, influence/power, and position within the schistosomiasis diagnostics landscape concerning the development of a device for improved diagnosis of schistosomiasis. Engaging and co-creating with stakeholders in diagnostic device development and adoption is known to be important for successful deployment and use of diagnostic devices. We improved upon an existing framework for stakeholder identification and applied it to the stakeholder identification process for co-creation. This framework can also be used to identify implementing stakeholders. We also analyzed relevant stakeholders' power, interest, and stakes for device co-creation using a power-interest matrix. This strategy will help to identify relevant stakeholders within the field of study and develop ways of engaging stakeholders based on the outcome of the analysis. To the best of our knowledge, this is the first study using a three-stage stakeholder approach to co-creation for a device for *S. haematobium*.

### Key Findings

Evidence from the analysis indicates two main uses of stakeholders: co-creation and adoption. It is also clear that some stakeholders fall into both the co-creation category and the implementation category. This is similar to what was found by van Limburg (50).

Among stakeholders for co-creation, most of the identified stakeholders within a formally organized system showed greater interest in the development of the device to either improve their work or increase efficiency. This suggests that the non-availability of point of care devices can impact on disease management of schistosomiasis. Although stakeholders at the community level had a low interest, this is likely due to low awareness of the disease, especially in its early stages or in cases of light infections (12, 42). Besides, the consequences of non-treatment are not probably clear to them due to the long time-to-complication seen in the disease (1, 2, 7).

The community-level stakeholders appear to demonstrate a low level of influence when analyzed individually. However, since the social power type was more common among these stakeholders, the stakeholders acting together can demonstrate a high level of power (26, 51, 52). For instance, they can decide not to allow the use of devices for testing within the community during large scale implementation. They can also refuse to go for testing based on their beliefs about the disease. As such, regardless of their low level of interests and power, it is important to keep them informed on device development processes such as prototypes for testing and as well as awareness campaigns that may precede device testing and adoption (29). Regular updates to the community will increase mobilization and buy-in, as well as the willingness to pay for schistosomiasis testing.

The most important type of stakeholders for our co-creation plan are the key players. These stakeholders demonstrated high levels of power by acting as key players within the health system (medical and organizational) and policy environment. These stakeholders are important for device co-creation and validation. The key players are important for strategizing and guiding product development. For instance, laboratory personnel can give insight to the peculiar challenges of equipment used within this context which may be different from the environmental context of the device developers. As expected, the financing/donor stakeholder has the highest level of power within stakeholders for co-creation because of the problems of financing healthcare and programs within the developing country context. It is well-known that donors strongly determine the direction of health policy within the context of Lower Middle-Income Countries (LMICs) (26). Engaging and working closely with these stakeholders will improve device design as well as increase acceptability by stakeholders who are important to adoption and implementation.

### Limitations

One limitation of our study was that we did not interview some stakeholders, for instance, political actors and media, who may be important for implementation, as well as the Federal Ministry of Health (FMOH) staff who may be important for co-creation in our interviews. However, these do not strongly influence the results of our work. It is known that the FMOH as a stakeholder is primarily involved in giving policy direction for schistosomiasis control and elimination (9). State governments are by law able to domesticate the policy and adopt what works for them by actively engaging with other non-state actors directly. Results of what works and progress on the schistosomiasis control program are usually reported to the FMOH. As such, we believe, we can leverage existing communication channels between the state and federal ministry to engage with stakeholders within the federal ministry during co-creation.

In respect of stakeholders for implementation, political actors especially were not interviewed because of the rapidly changing political landscape (52) in the state at the time of data collection and the long-life cycle of device development which creates problems with reengaging every new political actor throughout the device development lifecycle. Since co-creation is a major step in the life cycle of device development before the implementation phase, we believed that interacting with these co-creating stakeholders can increase our visibility within the healthcare context. Moreover, since some stakeholders are important for co-creation and implementation, our continuous engagement with these co-creating stakeholders would help to further identify other important stakeholders for implementation and adoption, as well as influence these implementing stakeholders (52). Finally, it is important to have a working prototype of the device first before involving other important implementers such as political actors and the media.

Another limitation is that some of our findings may not be generalizable to other parts of the country. Nigeria is a multi-ethnic society with ethnic groups concentrated in different regions. As such, the culture of the predominant ethnic group can affect how stakeholders interact with each other, how stakeholder roles are assigned, and the power dynamics within the schistosomiasis diagnostics landscape. For instance, in some parts of Nigeria, religious leaders may be a stakeholder within some communities. However, we believe this may not affect the result and the interpretation of the power-interest matrix for co-creation.

### Future Directions

In the future, we plan to further identify the value proposition of stakeholders for device development, as well as explore relationships between the stakeholders using social network analysis for both co-creation and implementing stakeholders. Identifying how stakeholders collaborate and communicate can aid in stakeholder engagement leveraging on the relationship ties to achieve mass acceptance and application of the diagnostic device.

## Data Availability Statement

The raw data supporting the conclusions of this article will be made available by the authors, without undue reservation.

## Ethics Statement

Ethical approval for the study was obtained from the Ethical Review Committee of the College of Medicine, University of Ibadan, Nigeria (NHREC/05/01/2008a). The patients/participants provided their written informed consent to participate in this study.

## Author Contributions

AO determined the overall structure of the study and the protocol and aligned it with the inputs from MK, OO, JD, and JV. AO analyzed the data with input from MK and OO. All authors reviewed the analyses, interpretation, reporting for critical content, and read and approved the final manuscript.

## Conflict of Interest

The authors declare that the research was conducted in the absence of any commercial or financial relationships that could be construed as a potential conflict of interest.
